# Proteomic Analysis of Copper Toxicity in Human Fungal Pathogen *Cryptococcus neoformans*

**DOI:** 10.3389/fcimb.2021.662404

**Published:** 2021-08-12

**Authors:** Tianshu Sun, Yanjian Li, Yingxing Li, Hailong Li, Yiyi Gong, Jianqiang Wu, Yating Ning, Chen Ding, Yingchun Xu

**Affiliations:** ^1^Medical Research Centre, State Key Laboratory of Complex Severe and Rare Diseases, Peking Union Medical College Hospital, Chinese Academy of Medical Science, Beijing, China; ^2^Beijing Key Laboratory for Mechanisms Research and Precision Diagnosis of Invasive Fungal Diseases, Beijing, China; ^3^College of Life and Health Sciences, Northeastern University, Shenyang, China; ^4^National Health Commission Key Laboratory of AIDS Immunology (China Medical University), National Clinical Research Center for Laboratory Medicine, The First Affiliated Hospital of China Medical University, Shenyang, China; ^5^Department of Clinical Laboratory, State Key Laboratory of Complex Severe and Rare Diseases, Peking Union Medical College Hospital, Peking Union Medical College, Chinese Academy of Medical Sciences, Beijing, China; ^6^Graduate School, Chinese Academy of Medical Sciences and Peking Union Medical College, Beijing, China

**Keywords:** *Cryptococcus neoformans*, copper toxicity, ROS, quantitative proteomics, ubiquitin degradation

## Abstract

*Cryptococcus neoformans* is an invasive human fungal pathogen that causes more than 181,000 deaths each year. Studies have demonstrated that pulmonary *C. neoformans* infection induces innate immune responses involving copper, and copper detoxification in *C. neoformans* improves its fitness and pathogenicity during pulmonary *C. neoformans* infection. However, the molecular mechanism by which copper inhibits *C. neoformans* proliferation is unclear. We used a metallothionein double-knockout *C. neoformans* mutant that was highly sensitive to copper to demonstrate that exogenous copper ions inhibit fungal cell growth by inducing reactive oxygen species generation. Using liquid chromatography-tandem mass spectrometry, we found that copper down-regulated factors involved in protein translation, but up-regulated proteins involved in ubiquitin-mediated protein degradation. We propose that the down-regulation of protein synthesis and the up-regulation of protein degradation are the main effects of copper toxicity. The ubiquitin modification of total protein and proteasome activity were promoted under copper stress, and inhibition of the proteasome pathway alleviated copper toxicity. Our proteomic analysis sheds new light on the antifungal mechanisms of copper.

## Introduction

*Cryptococcus neoformans* is a commonly inhaled fungal pathogen. It infects approximately 1 million individuals annually (Maziarz and Perfect, 2016) and causes over 181,000 deaths (Agustinho et al., 2018). Fifteen percent of AIDS-related deaths were reported to be associated with cryptococcosis. It also affects immunocompetent individuals (Datta et al., 2009; Kronstad et al., 2011; Andreou et al., 2020). The high mortality of cryptococcosis poses a huge burden, especially in medically deficient areas (Agustinho et al., 2018). Improved therapies are required to overcome challenges such as drug cost and availability, toxic side effects, lengthy treatment regimens, and resistance.

With the emergence of drug-resistant isolates, new antimicrobial agents, including metals, have received increasing attention. Copper (Cu), along with silver, gold, iron, zinc, and magnesium, has good antimicrobial properties (German et al., 2016; Vimbela et al., 2017). The *in vitro* antimicrobial properties of metals are mainly attributed to their electron transport function. Cu and Cu alloys are now widely used as antimicrobials in healthcare settings because of their “contact killing” antimicrobial activity. Cu was registered as the first solid antimicrobial material by the US Environmental Protection Agency (Grass et al., 2011). Copper can exist in two forms in cells: oxidized copper (Cu^2+^) or reduced cuprous (Cu^+^) (Gaetke and Chow, 2003). Several enzymes, such as lysyl oxidase, tyrosinase, cytochrome c oxidase, contain Cu, which acts as an electron donor/acceptor, alternating between oxidation and reduction (Karlin, 1993). However, the redox properties of Cu can also cause cell damage.

Metals also play an essential role in innate defense (Hood and Skaar, 2012). Copper aids in the host resistance against both bacteria and fungi (Li et al., 2019). Down-regulating the Cu transporter ATP7A on the lysosome and phagosome membranes was shown to inhibit the phagocytosis of bacteria and fungi by macrophages (White et al., 2009; Shen et al., 2018). After Cu supplementation, neutrophilic counts returned to normal in a patient with Coeliac disease (Khera et al., 2016). Ceruloplasmin, a multicopper oxidase, was elevated during infection with pathogens (Besold et al., 2016).

Pathogens have evolved complex Cu detoxification mechanisms to survive in their hosts. They can be divided into two aspects: Cu efflux systems and Cu sequestration (Chaturvedi and Henderson, 2014). *Mycobacterium tuberculosis* uses P-type ATPases (CtpC and CtpV) and the metallothionein MymT to resist Cu toxicity (Neyrolles et al., 2013). Defects in the Cu transporter CopA and the polycopper oxidase CueO in *Campylobacter jejuni* reduced its colonization of avian hosts (Gardner and Olson, 2018). *Candida albicans* adapts to high Cu levels by regulating the metallothioneins Cup1 and Crd2 through CaAce1/Cup (Ballou and Wilson, 2016). *C. neoformans* regulates Cu metabolism using Cuf1 to maintain Cu homeostasis (Ding et al., 2011; Jiang et al., 2011; Raja et al., 2013; Kosman, 2018). *C. neoformans* response to Cu stress was found to be activated upon lung invasion (Sun et al., 2014; Garcia-Santamarina and Thiele, 2015). Impairments in both metallothionein genes, *CMT1* and *CMT2*, reduce virulence during pulmonary infection (Ding et al., 2013; Ding et al., 2014). The membrane Cu importer *CTR4* in *C. neoformans* was down-regulated under Cu stress and it has been recognized as an indicator of Cu stress during pulmonary *C. neoformans* infections (Sun et al., 2014).

It has been reported that the toxicity of Cu to cells stems from its ability to induce reactive oxygen species (ROS) production (Gaetke and Chow, 2003). Cu^2+^ can be reduced to Cu^+^ in the presence of reducing agents such as superoxide, ascorbic acid, and glutathione (GSH). Cu^+^ catalyzes the conversion of hydrogen peroxide (H_2_O_2_) to form hydroxyl radicals (OH·) *via* the Haber–Weiss reaction (Bremner, 1998). The antimicrobial activity of Cu-induced ROS was reported to involves membrane breakdown, respiratory inhibition, protein inactivation, and DNA degradation (Hans et al., 2015). Cu toxicity in *C. neoformans* has been investigated at the transcriptome level (Garcia-Santamarina et al., 2018), but little is known about how Cu modulates protein expression in fungal cells. In this study, we investigated the antifungal action of Cu by assessing proteomic changes using liquid chromatography-tandem mass spectrometry (LC-MS/MS).

## Methods

### Strains and Medium

*Cryptococcus neoformans* var. *grubii* (serotype A) H99, was used as the wildtype strain for the experiment. *cmt1/2ΔΔ* is a mutant *C. neoformans* strain on wild-type strain H99. The mutant is generated by knocking out both *CMT1* and *CMT2*, which encode metallothioneins, with the selection markers being *NAT* and *NEO* (Ding et al., 2013). The cells were routinely grown in yeast extract peptone dextrose medium (YPD: 1% yeast extract, 2% peptone, and 2% dextrose). CuSO_4_ (final concentrations: 0.5 or 1 mM) was added to induce Cu stress. N-acetylcysteine (NAC; final concentrations: 30, 40, or 50 mM) was added to block ROS production. Colony images were captured using ImageQuant LAS 500 (GE, Boston, MA, USA).

### ROS Measurement

Intracellular ROS levels were measured using a ROS assay kit (CA1410; Solarbio, Beijing, China). Overnight cultures of *cmt1/2ΔΔ* were diluted in fresh YPD medium to an optical density at 600 nm of 0.2. The cells were treated with 0.5 or 1mM Cu at 30 °C for 4 h (n=3). The cells were then collected and washed twice in phosphate-buffered saline (PBS). Next, about 2×10^7^ cells were stained with 20 μM 2′,7′-dichlorofluorescin diacetate (DCFH-DA) for 1 h. DCFH-DA (non-fluorescent) was hydrolyzed to dichlorofluorescin (DCFH) after entering cells. When DCFH is in the presence of ROS, it is oxidized to dichlorofluorescein (DCF), which is a strong green fluorescent substance. Fluorescence intensity was measured using microplate luminometers (Varioskan Flash; Thermo Fisher Scientific, Waltham, USA), with excitation at 488 nm and emission at 525 nm. The intracellular ROS level is shown as fluorescence value divided by the absorbance at 600 nm.

### Real-Time PCR

*cmt1/2ΔΔ* was cultured in YPD medium and treated with 0.5 mM CuSO_4_ or both 0.5 mM CuSO_4_ and 30 mM NAC (n=3). RNA samples used for real-time PCR were isolated using TRIzol reagent (Thermo Fisher Scientific, Waltham, MA, USA) followed by TURBO DNase I treatment (Thermo Fisher Scientific) to eliminate DNA contamination. One microgram of total RNA was reverse-transcribed into cDNA using the GoScript Reverse Transcription System (Promega, Madison, WI, USA). Real-time PCR was performed using a CFX Connect thermal cycler (Bio-Rad, Hercules, CA, USA). The data were analyzed using the 2*^−ΔΔCt^* method. *ACT1* was used as the loading control. **Table S1** lists the primer pairs used.

### Protein Extraction, Digestion, and Labeling

*cmt1/2ΔΔ* was cultured in YPD medium and treated with 0.5 mM Cu or both 0.5 mM Cu and 30 mM NAC. The cells were washed twice in PBS and proteins were then extracted using lysis buffer (7 M urea, 2 M sulfourea, and 0.1% CHAPS) with a protease inhibitor cocktail using a Precellys Evolution tissue homogenizer (Bertin, Montigny-le-Bretonneux, France). Protein concentrations were determined using the Bradford method. Dithiothreitol was added to a 100 µg protein extract to a final concentration of 25 mM, and the mixture was incubated at 37°C for 1 h. Iodoacetamide (163-2109; Bio-Rad) was then added to a final concentration of 50 mM and the mixture was incubated at room temperature in the dark for 30 min. Next, the mixture was sieved through a 10-kDa filter (UFC501096; MilliporeSigma, Burlington, MA, USA) and washed thrice in 0.2 M triethylammonium bicarbonate (TEAB, T7408, MilliporeSigma) buffer. The protein was digested overnight with 2 μg trypsin (trypsin to protein mass ratio of 1:50) at 37°C. Next, the peptides were labeled using isobaric tags for relative and absolute quantitation (iTRAQ) reagents (4381663; AB Sciex, Framingham, MA, USA) desalted using a hydrophilic/lipophilic balanced column (WAT094226; Waters, Milford, MA, USA), and vacuum-dried. The iTRAQ-labeled peptides were dissolved in 2% acetonitrile, and then fractionated using high-pH high-performance liquid chromatography (HPLC; Xbridge Peptide BEH C18 column, 4.6 mm × 150 mm, 3.5 μm; Waters). The elution gradient was 95–5% phase A (2% acetonitrile, pH 10) and 5–95% phase B (98% acetonitrile, pH 10) for 60 min. The eluted peptides were collected at a rate of one fraction per min. To shortening the loading time in the LC-MS/MS analysis, 60 offline fractions were pooled into 10 fractions and lyophilized. All fractions were then combined for database searching.

### LC-MS/MS Analysis

Dried peptides were re-suspended in 0.1% formic acid and loaded onto a reversed-phase C18 analytical column (75 μm × 150 mm, 3 μm, custom-made in our lab). Elution was accomplished using a constant flow (0.6 μL/min) of buffer A (0.1% formic acid) and buffer B (80% acetonitrile) over a gradient from 5% to 38% for 120 min. The polypeptide mixture was analyzed using an Orbitrap Q-Exactive Plus mass spectrometer (Thermo Fisher Scientific). Each full scan was a high-speed signal-dependent scan. The first-level full scan was performed from 350 to 1,800 *m/z* at 70,000 resolution, and automatic gain control was set at 3e6. The second-level scan was performed from 100 to 1,800 *m/z* at 17,500 resolution, and automatic gain control was set at 1e5. Higher-energy collisional dissociation mode was used, with a normalized collision energy of 32% and a scanning time of 120 min.

### Database Search and Bioinformatics Analysis

The LC-MS/MS data were searched against the UniProt *Cryptococcus neoformans* H99 database (https://www.uniprot.org/proteomes/UP000010091), which provide resource of protein sequence and functional information, using Mascot (version 2.5.1) and Scaffold (version 4.6.2) software, assuming the digestion enzyme trypsin and two missed cleavages at maximum. The MS tolerance was 10 ppm and the fragment ion mass tolerance was 0.02 Da. Carbamidomethyl of cysteine and iTRAQ-8plex of lysine and the n-terminus were specified in Mascot as fixed modifications. Oxidation of methionine, acetyl of the n-terminus and iTRAQ-8plex of tyrosine were specified in Mascot as variable modifications. Protein identifications were accepted as contained at least 2 identified peptides. Protein probabilities were assigned by the Protein Prophet algorithm (Nesvizhskii et al., 2003).

Proteins were considered to be differentially expressed if the ratio <0.769 or >1.3 (foldchange > 1.3) (Zhu et al., 2020; Jiang et al., 2020) and FDR corrected *q*-value <0.05. The quantified proteins were annotated by Gene Ontology (GO) and Kyoto Encyclopedia of Genes and Genomes (KEGG) analyses. GO analysis was performed using clusterProfiler (version 3.14.0) in R (version 3.6.1) (Yu et al., 2012). *C. neoformans* gene ontology data were downloaded from Annotation Hub. The EnrichGO and compareCluster functions were used for the analyses. KOBAS (version 3.0) was employed to perform the KEGG analysis (Li et al., 2019).

### Parallel Reaction Monitoring Assay

PRM, a reliable targeted LC-MS analysis, was used as a validation of the quality of iTRAQ-proteomic data in this research, considering antibodies to target proteins were not easy to be acquired in *C. neoformans*. It can provide higher accuracy, sensitivity and reproducibility data compared with non-targeted profile (Manes and Nita-Lazar, 2018). The proteins were reduced with 0.25 M dithiothreitol, alkylated with 0.5 M iodoacetamide, cleaned thrice with 0.2 M TEAB, digested overnight with trypsin (the ratio of trypsin to protein was 1:50), then cleaned thrice with 0.5 M TEAB and collected by centrifugation. Solid-phase extraction was performed using a ZipTip C18 column (Millipore, USA). After separation using an EASY-nLC liquid phase, the proteins were subjected to low-pH reversed-phase C18 capillary chromatography (150 μm×150 mm, 1.9 μm). Phase A consisted of 0.1% formic acid and the phase B consisted of 80% acetonitrile and 0.1% formic acid. The elution gradient was 13–38%, the total elution time was 60 min, and the flow rate was 0.6 μL/min. The polypeptide mixture was identified using an Orbitrap Q-Exactive Plus mass spectrometer. High-sensitivity and data-dependent acquisition scanning modes were used, and the scanning time was 120 min. The first-level full scan was performed from 300 to 1500 *m/z* at 60,000 resolution, with collision energy of 30%. The second-level scan was performed at 15,000 resolution. PRM was conducted according to the data-dependent acquisition scanning results. Each full scan was followed by 25 targeted scans, and the scanning time was 60 min. The first-level full-scan was performed from 300 to 1250 *m/z* at 60,000 resolution and automatic gain control was set at 3e6, with a normalized collision energy of 28% and a maximum injection time of 80 ms. The resolution of the secondary scan was 30,000. The mixed essential-spectral data were retrieved by Proteome Discoverer (version 2.1). The retrieval parameters were obtained from the UniPort *Cryptococcus neoformans* H99 database. Mass errors of parent and fragmentation ions were 10 ppm and 0.05 Da, respectively. Quality control parameters included polypeptide false discovery rate <1.0% and protein false discovery rate <1.0%, with each protein identified to at least one specific polypeptide. Pooled peptide samples (2 μg of each sample) were subjected to LC-MS/MS analysis for preparation of the spectrum library. Skyline (version 4.1.1.11725) was used to process the MS data.

### Western Blot

*cmt1/2ΔΔ* was treated with 0.5 mM Cu in YPD medium at 30°C for 4 h (n=3). The cells were washed twice in cold PBS, and then the proteins were extracted in lysis buffer (7 M urea, 2 M sulfourea, and 0.1% CHAPS) with a proteinase inhibitor and phenylmethylsulfonyl fluoride (PMSF). Next, 30 µg of protein was used for sodium dodecyl sulfate-polyacrylamide gel electrophoresis. Western blot assays were performed using an anti-ubiquitin antibody (1:1,000; 3933; Cell Signaling Technology, Boston, MS, USA), anti-histone H3 antibody (1:1,000; 4499S; Cell Signaling Technology), and goat anti-rabbit IgG (H+L) secondary antibody (1:5,000; A16096; Thermo Fisher Scientific). The results were imaged using ChemiDoc XRS+ (Bio-Rad, USA).

### Proteasome Activity Assay

*cmt1/2ΔΔ* was treated with 0.5 mM Cu, 0.5 mM Cu and 30 mM NAC, or 1 mM Cu in YPD medium at 30°C for 4 h (n=3). Cells were washed twice in cold PBS, and then lysed using in 0.5% NP40. Proteasome activity was assessed using a proteasome activity assay kit (ab107921; Abcam, Cambridge, MA, USA). The samples were mixed with proteasome substrate and incubated at 37°C. The proteasome activity units (relative to the standard fluorescence intensity) were determined according to the fluorescence consumed from 30 to 60 minutes of the reaction. Fluorescence intensity was measured using a multi-mode reader (SYNERGY-LX; BioTek, Winooski, VT, USA), with excitation at 350 nm and emission at 440 nm.

### Growth Kinetics Assay

*cmt1/2ΔΔ* was shake cultured in YPD medium overnight at 30°C. The cells were then treated with 0.5 mM Cu, 10 μg/mL proteasome inhibitor MG132 (HY-13259; MedChemExpress, Monmouth Junction, NJ, USA), or both 0.5 mM Cu and 10 μg/mL MG132 in 96-well plates (n=3). The optical density at 600 nm of the initial culture system was 0.05. The optical density of the cells was detected every 4 h to plot the growth curves.

### Statistics and Reproducibility

Statistical analysis of ROS level, real-time PCR data, and proteasome activity data was performed in GraphPad Prism 6.0 software. Significant differences between two groups were determined by Student’s t-test (*p values<0.05, **p values<0.01, ***p values<0.005). comparison between multiple treatments was performed using ANOVA (***p values<0.005). All experiments were performed using three biological replicates to ensure reproducibility. Proteomic data reproducibility was analyzed using principal component analysis (PCA), using the R package ggplot2 (version 3.2.1). Two-tailed Fisher’s exact tests were used to assess the GO and KEGG enrichment of the differentially expressed proteins against all identified proteins.

### Data Availability

The mass spectrometry proteomics data have been deposited to the ProteomeXchange Consortium (http://proteomecentral.proteomexchange.org) *via* the iProX partner repository with the dataset identifier PXD024098 http://proteomecentral.proteomexchange.org/cgi/GetDataset?ID=PXD024098.

## Results

### Exogenous Cu Induces Intracellular ROS Generation in *C. neoformans*

**Figure 1:**
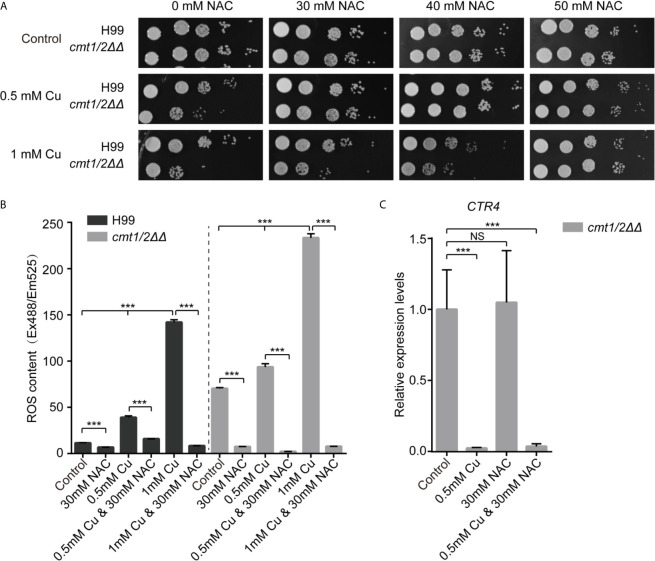
N-acetylcysteine reduces the ROS-toxicity induced by copper in *Cryptococcus neoformans*. **(A)** Growth of *C neoformans* following treatment with Cu and N-acetylcysteine (NAC). Clinically isolated wild-type *C. neoformans* strain H99 and the Cu-sensitive mutant *cmt1/2ΔΔ* were grown in yeast extract peptone dextrose (YPD) liquid medium overnight, serially diluted and cultured on agar medium at 30°C for 2 days. **(B)** Levels of intracellular ROS following treatment with Cu and NAC. Cells were treated with 0.5 or 1mM Cu and NAC was added to scavenge ROS. The intracellular ROS level is shown as fluorescence intensity (Ex488/Em525) divided by the absorbance at 600 nm. ***p-value <0.005 (*n*=3). **(C)** Relative expression of the Cu importer *CTR4* in the Cu-sensitive mutant *cmt1/2ΔΔ*. Cells were treated with 0.5 mM Cu and/or 30 mM NAC and RNA was extracted and analyzed by real-time polymerase chain reaction. ***p-value <0.005 (*n*=3). *NS*, not significant.

It was reported that the antimicrobial activity of Cu is associated directly with its oxidative characteristics (Vincent et al., 2018). The activities of superoxide dismutase, catalase and glutathione peroxidase, proteins associated with ROS metabolism, in Cu sensitive strain *cmt1/2ΔΔ* were improved under copper stress (Sun et al., 2021). But whether ROS is the primary cause of cell death was unknown. We used N-acetylcysteine (NAC), an aminothiol and synthetic precursor of intracellular cysteine and glutathione, as an ROS scavenger. It is a sulfhydryl-containing antioxidant that increases the free radical scavenging in cells. The metallothioneins double-knockout mutant *cmt1/2ΔΔ* is very sensitive to Cu stress compared with wildtype strain H99, which was consistent with previous job (Ding et al., 2013), but it did not exhibit growth retardation when NAC was added to the medium. The growth of H99 and *cmt1/2ΔΔ* were not affected by NAC treatment alone. With an increased Cu concentration, more NAC was required to neutralize the effects of Cu stress. When the Cu concentration was 0.5 mM, 30 mM NAC was required to prevent toxicity, and when it was raised to 1mM, 50 mM NAC was required to prevent toxicity (**Figure 1A**). This preliminary experiment suggested that the generation of intracellular ROS is an important factor in Cu toxicity, although ROS-independent Cu toxicity remains possible (e.g., impairment of iron–sulfur protein biogenesis) (Brancaccio et al., 2017).

We used DCFH-DA fluorescent probes to image intracellular ROS under Cu stress, and we found a positive correlation between Cu concentration and intracellular ROS. Under Cu stress, *cmt1/2ΔΔ* (which was deficient in the Cu detoxification factors *CMT1* and *CMT2*) generated more ROS than the wild-type strain H99. However, Cu-induced intracellular ROS in both the wild-type and *cmt1/2ΔΔ* strains was suppressed by NAC (**Figure 1B**). To assess whether NAC operates independent of chelating Cu ions during the Cu detoxification process, we assessed the expression of the membrane Cu importer *CTR4*, which is down-regulated under Cu stress, indicating high Cu concentration sensed by cells (Sun et al., 2014). *CTR4* was down-regulated in *cmt1/2ΔΔ* under Cu treatment, regardless of whether NAC was added to the medium (**Figure 1C**). This indicated that the NAC action was attributable to its antioxidant activity rather than depletion of Cu ions.

### Quantitative Proteomic Profile of *C. neoformans* Under Cu Stress

**Figure 2:**
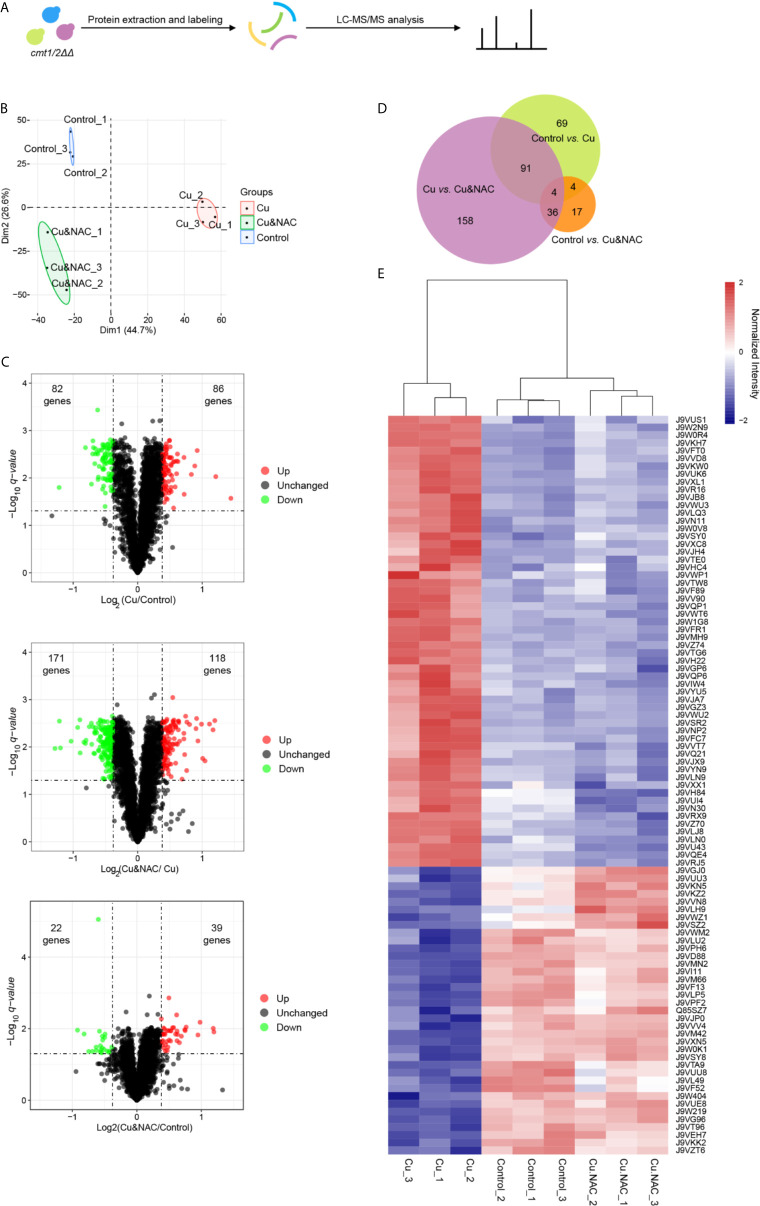
iTRAQ quantitative proteomic analysis of the copper (Cu)-sensitive mutant *cmt1/2ΔΔ* following treatment with Cu or Cu and N-acetylcysteine (NAC). **(A)** Study flow diagram. The metallothionein double-knockout strain *cmt1/2ΔΔ* was grown in yeast extract peptone dextrose (YPD) liquid medium overnight and divided into three groups: an untreated (Control) group (*n*=3), a group exposed to Cu (*n*=3), and a group exposed to both Cu and NAC (*n*=3). Cells grown to the logarithmic stage were lysed to extract proteins, which were digested into peptides, labeled, and assessed by liquid chromatography-tandem mass spectrometry (LC-MS/MS). **(B)** Principal component analysis (PCA) of proteomic data. Raw proteomic data were obtained using LC-MS/MS and analyzed using the R package ggplot2 (version 3.2.1). Each point in the figure represents a single sample and treatment groups are distinguished by color. **(C)** Volcano plots of differentially expressed proteins. Each point represents a protein. The x-axis represents the log2 transformation of the protein ratio between two groups. The y-axis represents the −log10 transformation of the FDR corrected *q*-values of the comparisons between the two groups. Green dots represent down-regulated proteins, red dots represent up-regulated proteins, and black dots represent unchanged proteins. **(D)** Venn diagram of differentially expressed proteins in the three pairs of comparisons (Cu *vs.* Control, Cu&NAC *vs.* Cu and Cu&NAC *vs.* Control). The size of each circle represents the number of differentially expressed proteins. **(E)** Heatmap of differentially expressed proteins common to the two comparisons. The x-axis represents samples, and the y-axis represents proteins. The colors represent protein expression levels in the sample: the maximum value is red, and the minimum value is blue. According to the similarity of color between samples, the proteins were divided into two clusters: up-regulated under Cu stress and down-regulated under Cu stress.

Based on our previous work and the data presented in **Figure 1** showing overall concordance regarding the responses to Cu and NAC between wild-type and *cmt1/2ΔΔ* strains, we anticipated that Cu-sensitive *cmt1/2ΔΔ* would accurately reflect the overall response of *C. neoformans* to Cu stress, but with a magnitude of changes more readily detectable at the proteomic level. We therefore used *cmt1/2ΔΔ* to amplify the Cu stress response of *C. neoformans* in the whole proteome analysis. The cells were cultured to the logarithmic phase and then collected for protein extraction. To investigate the function of ROS in response to Cu treatment, the ROS scavenger NAC was added to the medium (**Figure 2A**). A total of 3,529 proteins were quantified by LC-MS/MS (**Table S2**) and validated for mass accuracy (**Figure S1**). Principal component analysis suggested that the exposure groups could be differentially separated into three clusters (**Figure 2B**). In the cells treated with Cu alone compared to untreated cells, 168 proteins were differentially expressed. In the cells treated with NAC plus Cu compared to Cu alone, 289 proteins were differentially expressed. In the cells treated with NAC plus Cu compared to untreated cells, 61 proteins were differentially expressed (ratio < 0.769 or> 1.3, FDR corrected *q*-value <0.05) (**Figure 2C** and **Table S3**). Among the two sets of differentially expressed proteins in control *vs.* Cu and Cu *vs.* Cu&NAC comparisons, 95 proteins overlapped, and these were considered potential targets of Cu-induced ROS (**Figure 2D**). Though 4 proteins were overlapped in the 3 pairs of comparisons, they all showed the different trend in control *vs.* Cu compared with control *vs.* Cu&NAC. The expression of the 95 proteins (37 down-regulated and 58 up-regulated under Cu stress) was normalized by NAC (**Figure 2E**). To verify the reliability of the iTRAQ quantitative proteomic analysis, 82 peptides (**Table S4A**) of 37 proteins that were notably differentially expressed and involved in the enrichment pathways were selected and measured by PRM (Yu et al., 2012). The target LC-MS/MS results were consistent with the iTRAQ-LC-MS/MS analysis for 35 of the 37 proteins (ratio < 0.769 or > 1.3, FDR corrected *q*-value <0.05) (**Table S4B**). Most of the proteins identified were on the regression line in the correlation plot (**Figure S2**). The two unverified proteins, have not been found differentially expressed, may be limited by the reliability of the representative peptide segments.

### Gene Ontology Analysis of Differentially Expressed Proteins Under Cu Stress

**Figure 3:**
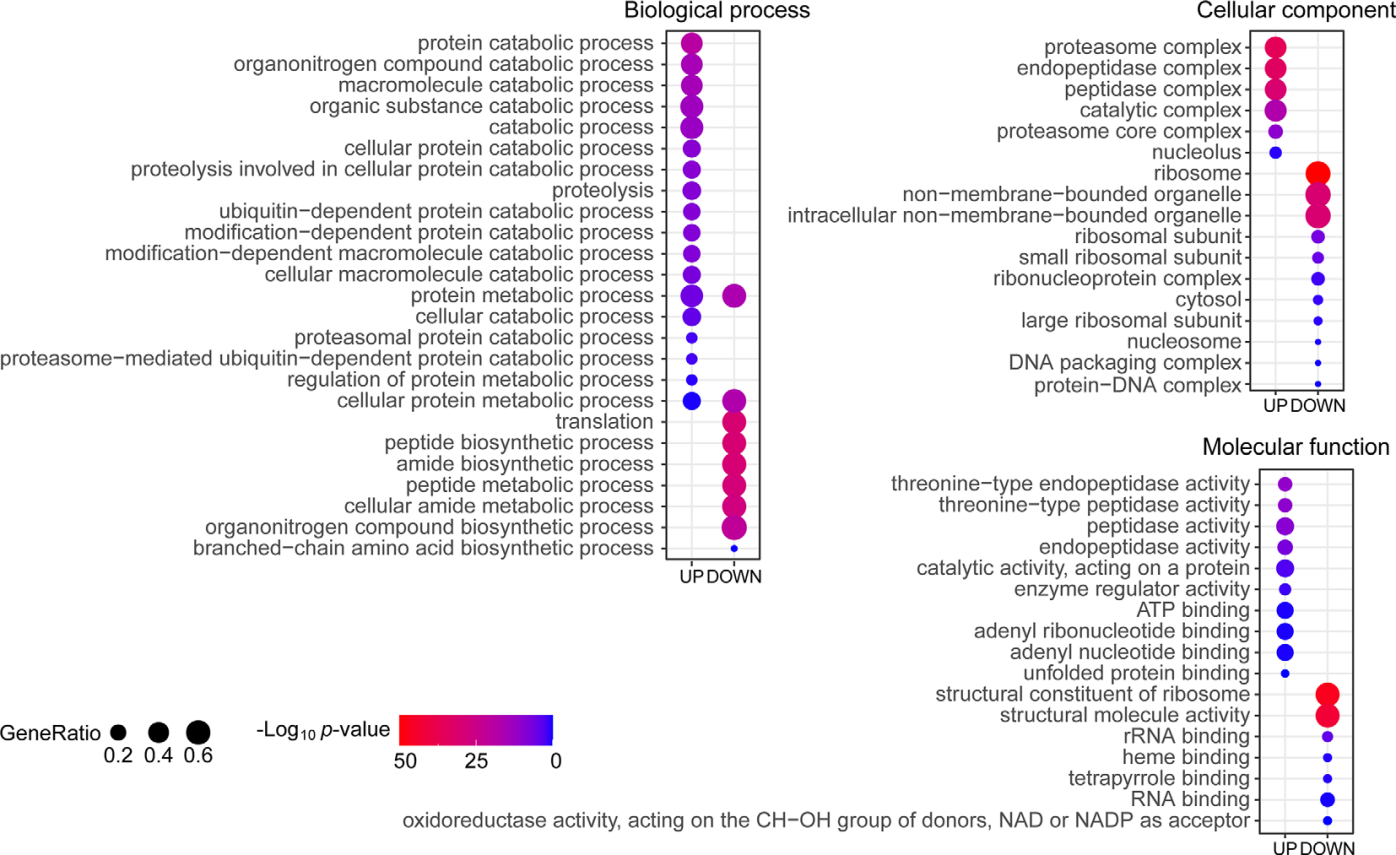
Gene Ontology (GO) enrichment of differentially expressed proteins under copper (Cu) stress. The proteins were annotated with subcategory terms in the three GO categories: biological processes, cellular compartments, and molecular functions. For each category, a two-tailed Fisher’s exact test was used to assess the enrichment of the differentially expressed protein against all identified proteins. The GO subcategories with a *p*-value <0.05 were considered significantly enriched and shown in the figure. The size of the bubbles represents the proportion of differentially expressed proteins in this subcategory among the total number of proteins in the corresponding category.

To globally analyze the molecular mechanism of Cu toxicity, based on the main functions of the differentially expressed proteins, we performed GO annotation using Blast2GO software (version 4.1.9) (Gotz et al., 2008) followed by enrichment analysis using the R cluster Profiler R package. The GO items with a *p*-value <0.05 were enriched in the biological process, cellular component, and molecular function categories from identified up-regulated proteins and down-regulated proteins under Cu stress, respectively (Ashburner et al., 2000). The up-regulated proteins were enriched in protein catabolic processes and ubiquitin-dependent protein catabolic processes. These changes mainly occurred in the proteasome complex, endopeptidase complex, peptidase complex, and catalytic complex, involving molecular functions related to endopeptidase activity, peptidase activity, catalytic activity acting on proteins, enzyme regulator activity, ATP binding and nucleotide binding. The down-regulated proteins were enriched in protein metabolic processes, translation, peptide biosynthetic process, amide biosynthetic process, organonitrogen compound biosynthetic process and branched-chain amino acid biosynthetic process. These changes mainly occurred in the ribosome, non-membrane-bound organelles, involving molecular functions related to structural constituents of ribosome, structural molecule activity and RNA binding (**Figure 3**). This indicated that the main features of the Cu toxicity were decreased in protein synthesis and increased protein degradation.

### Quantile-Based Clustering Analysis

**Figure 4:**
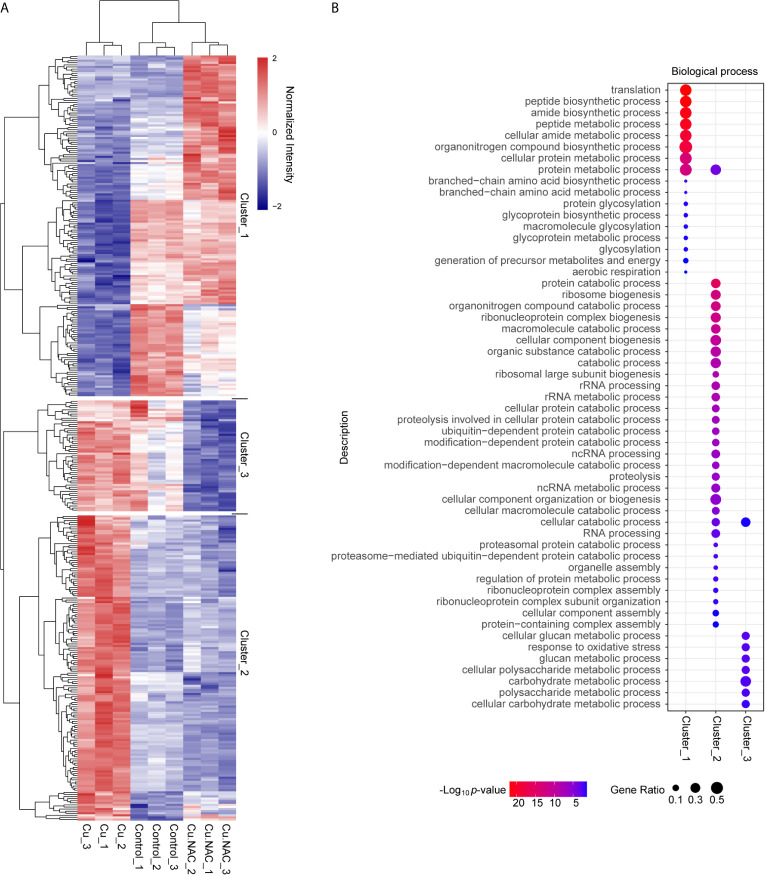
Clustering-based Gene Ontology (GO) enrichment analysis of differentially expressed proteins following treatment with copper (Cu), Cu and N-acetylcysteine (NAC), or untreated (control). **(A)** Hierarchical clustering analysis of differentially expressed proteins under Cu stress. The x-axis represents the clustering of the samples and the y-axis represents the clustering of the differentially expressed proteins. The colors represent the relative expression levels of the proteins: the maximum value is red and the minimum value is blue. **(B)** Bubble diagram of enriched GO subcategories for the differentially expressed proteins in the three clusters, indicating *p*-values and protein ratios.

To clearly delineate the changes in protein expression in each treatment group, we constructed a heatmap of differentially expressed proteins (**Figure 4A**). The proteins were clustered according to their global expression characteristics and z-scores were calculated according to the normalized intensity in the horizontal and vertical dimensions separately. These z-scores were then clustered by one-way hierarchical clustering (Euclidean distance, average linkage clustering) using Genesis. Cluster membership was visualized using the “heatmap.2” function from the “gplots” package in R. As expected, the expression of proteins from cells treated with Cu plus NAC exhibited overall similarities to control cells, though the expression of some proteins was more similar to that in cells treated with Cu alone. Cu-treated cells also exhibited some unique overlap with control cells that differed from the cells treated with Cu plus NAC, which may be partly attributable to the detoxification role of NAC in Cu stress.

Based on further hierarchical clustering, the differentially expressed proteins were divided into three clusters according to their distinctive expression characteristics. For each cluster, we grouped all the enriched functional categories along with their *p*-values (**Figure 4B**). The proteins in cluster 1 were down-regulated under Cu stress but not when Cu was administered with NAC; these proteins were mainly involved in biological processes related to translation, peptide synthetic process, amide synthetic process, peptide metabolic process, organonitrogen compound synthetic process and protein metabolic processes. The proteins in cluster 2 were up-regulated under Cu stress but not when Cu was administered with NAC; these proteins were mainly involved in protein catabolic process, ribosome biogenesis, organonitrogen compound catabolic process, ribonucleoprotein complex biogenesis, macromolecule catabolic processes, cellular compound biogenesis, organic substance catabolic process, rRNA processing, ubiquitin-dependent protein catabolic processes, ncRNA processing, cellular component organization or biogenesis, and protein metabolic process. The proteins in cluster 3 were down-regulated when Cu was administered with NAC; these proteins were related to glucan metabolism processes, response to oxidative stress, polysaccharide metabolic processes, carbohydrate metabolic processes and cellular catabolic process. In general, biological processes related to cluster 1 and cluster 2 proteins were the targets of ROS-dependent Cu toxicity.

### Ubiquitin-Mediated Proteolysis Is Up-Regulated in *C. neoformans* in Response to Cu Stress

**Figure 5:**
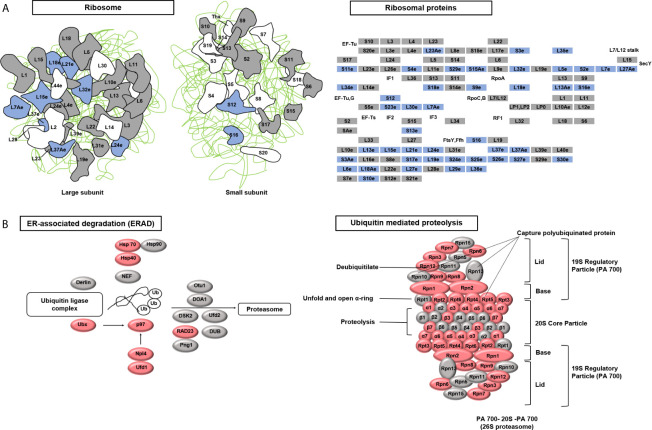
Kyoto Encyclopedia of Genes and Genomes (KEGG) pathways targeted after copper (Cu)-induced accumulation of reactive oxygen species (ROS). **(A)** The ribosome pathway was down-regulated under Cu stress. The KEGG database was used to identify enriched pathways. A schematic diagram of ribosome subunits from the KEGG database is shown. All proteins in the ribosome pathway that were identified in the proteomic analysis are displayed in squares. Blue indicates down-regulated proteins (*q*-value <0.05) and gray indicates unchanged proteins. **(B)** Two protein degradation pathways (endoplasmic reticulum-associated degradation and ubiquitin-mediated proteolysis) were up-regulated under Cu stress. All proteins identified in these two pathways that were identified in the proteomic analysis are displayed in ellipses. Red indicates up-regulated proteins (*q*-value <0.05) and gray indicates unchanged proteins.

To identify the target pathways under Cu stress, we conducted a KEGG enrichment analysis in KOBAS (version 3.0) (Kanehisa et al., 2012). The proteins that were differentially expressed in cells treated with Cu alone compared to untreated control cells were enriched in three pathways: ribosome, proteasome, and protein processes in the endoplasmic reticulum. The differentially expressed proteins included ribosome and proteasome subunits, chaperones (HSP70 and HSP90), and glycogen metabolism-related protein (Ubx), ubiquitin-conjugating enzyme variant MMS2 (p97), ER-associated protein catabolism-related protein (Npl4), ubiquitin fusion-degradation 1-like protein (Ufd1), and ubiquitin receptor (RAD23) (**Table S5**). Under Cu stress, 37% of the ribosomal subunits were down-regulated, while 66% of the ubiquitin ligase complex and proteasomal subunits were up-regulated (**Figure 5**). Cells treated with Cu plus NAC compared to untreated control cells could prevent from these changes in ribosomal subunits. These findings suggest that Cu-induced ROS alter protein synthesis and degradation processes.

**Figure 6:**
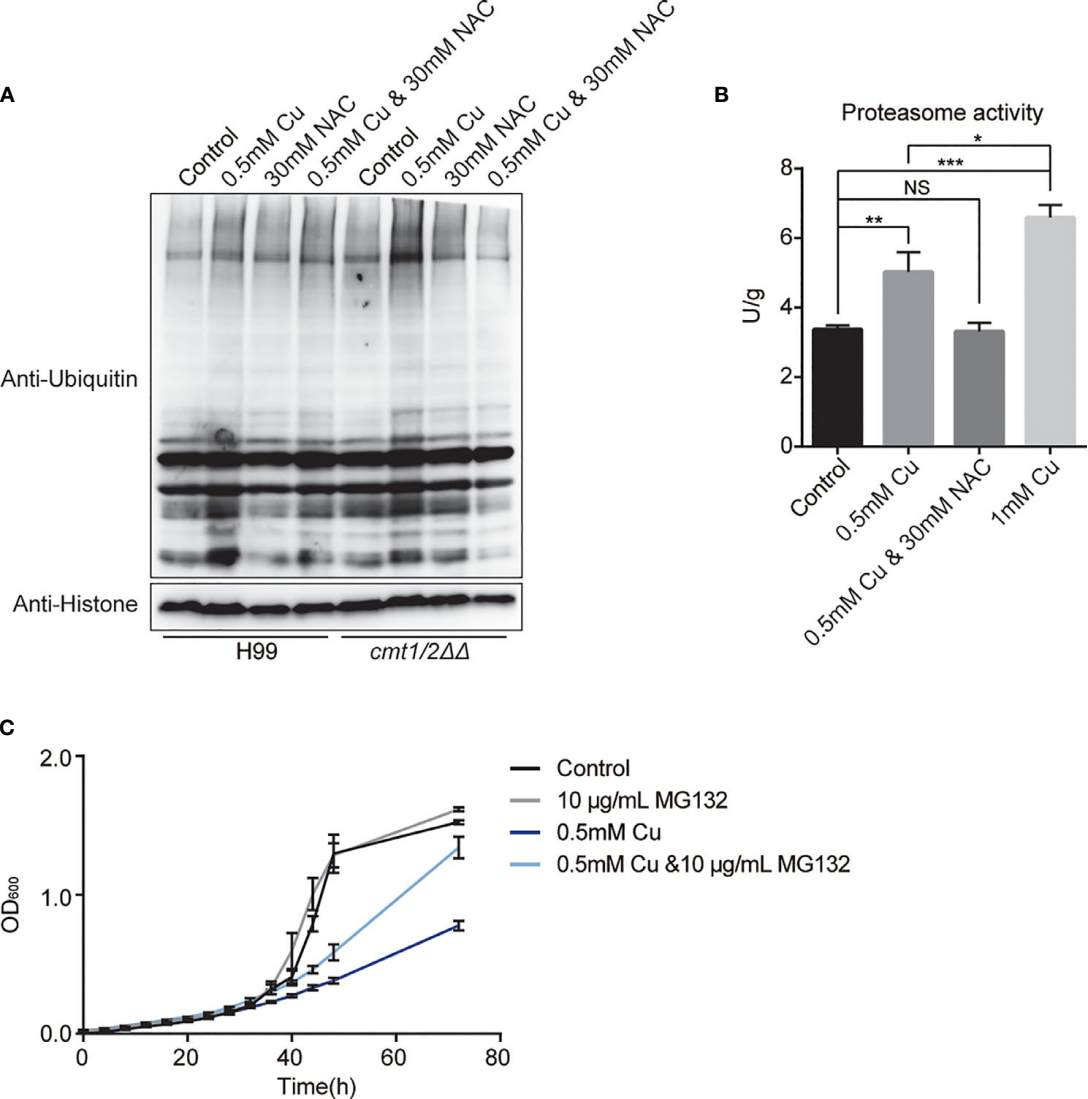
Ubiquitin-mediated proteolysis under copper (Cu) stress. **(A)** Western blot analysis of ubiquitinated proteins in the wild-type strain H99 and Cu-sensitive mutant *cmt1/2ΔΔ.* Cells were treated with 0.5 mM Cu, 30mM NAC,0.5 mM Cu plus 30mM NAC, respectively in yeast extract peptone dextrose (YPD) medium. The grayscale of the bands is positively correlated with the relative expression of ubiquitination level of total proteins. **(B)** Proteasome activity assay of *cmt1/2ΔΔ* under Cu stress. *cmt1/2ΔΔ* mutant was induced by 0.5 mM Cu, 1 mM Cu, or both 0.5 mM Cu and 30 mM N-acetylcysteine (NAC) in YPD medium for 4 h. *p-value <0.05, **p-value <0.01, ***p-value <0.005 (n=3). NS, not significant. The fluorescence of the samples was quantified using the standard substance in the proteasome activity assay kit. Proteasome activity is shown in U/g protein. Histone was used as an internal reference. **(C)** Growth (based on optical density at 600 nm) of *cmt1/2ΔΔ* under Cu stress was affected by the proteasome inhibitor MG132. *cmt1/2ΔΔ* was incubated in YPD medium with or without 0.5 mM Cu and 10 μg/mL MG132 (n=3).

Western blotting showed that Cu stress up-regulated the total ubiquitination in *cmt1/2ΔΔ* (**Figure 6A**). The proteasome activity assay of *cmt1/2ΔΔ* indicated increased proteasome activity after treatment with 0.5 or 1 mM Cu, but unchanged when treated with both 0.5 mM Cu and 30 mM NAC (**Figure 6B**). The proteasome inhibitor MG132 partially rescued the *cmt1/2ΔΔ* growth inhibition after Cu treatment (**Figure 6C**), without changing the cell size and morphology (**Figure S3**) It indicates that promoting ubiquitin-mediated proteolysis is a primary effect of Cu toxicity.

## Discussion

The toxicity of Cu to microbial pathogens has been used as a host defense strategy (Wiemann et al., 2017; Shen et al., 2018). In particular, Cu toxicity affects the virulence of *C. neoformans* during pulmonary infection (Ding et al., 2013), although the exact mechanism has not been fully elucidated. Cu can exist in an oxidized or reduced state, influencing its catalytic activity and contributing to ROS production (Kaur et al., 2019). We confirmed that intracellular ROS accumulated under Cu stress, especially in a Cu-sensitive mutant that lacked metallothioneins. We hypothesize that Cu toxicity in *C. neoformans* was ROS-dependent, as cell growth under Cu stress on plated media was not affected in the presence of a ROS inhibitor.

The Cu metabolic pathway is well understood in fungi (Smith et al., 2017; Garcia Silva-Bailao et al., 2018; Li et al., 2019; Raffa et al., 2019; Antsotegi-Uskola et al., 2020), but the molecular mechanisms underlying Cu toxicity have mainly been explored in bacteria. It was postulated that the targets of Cu toxicity are nucleic acids, structural and functional proteins, lipids, and inhibition of metabolic processes such as respiration and osmotic pressure, leading to cytolysis (Gaetke et al., 2014; Tan et al., 2017). To investigate the primary intracellular targets of Cu-induced ROS, we compared the proteomic profiles of a Cu-sensitive metallothionein double-knockout mutant among the Cu treatment, Cu plus NAC treatment groups, and control groups. The differentially expressed proteins under Cu stress were restored by NAC, an ROS scavenger, suggesting that ROS accumulation is a key factor in Cu toxicity.

We investigated the normalized intensity identified by MS of several proteins associated with ROS metabolism (**Figure S4**) (Aguirre et al., 2006). For example, Sod2 (J9VWW9_CRYNH) was up-regulated during Cu treatment, but not in the presence of NAC, while Sod1 (J9VLJ9_CRYNH) was stably expressed under each treatment. These results were consistent with those reported in *Histoplasma*: Sod2 protects yeasts specifically from exogenous superoxide, while intracellular Sod1 eliminates endogenous ROS (Youseff et al., 2012). In addition, Catalase3 and peroxidase Tsa1 (J9VH55_CRYNH) were significantly up-regulated under Cu treatment. Furthermore, Cu up-regulated the iron-sulfur cluster transporter Atm1 (J9VWU3_CRYNH) and iron-sulfur protein assembly co-chaperone HscB (J9VWP1_CRYNH). These results were consistent with the previous research (Aguirre et al., 2006; Perez-Gallardo et al., 2013; Garcia-Santamarina et al., 2017)

The role of Cu in DNA damage is controversial. Macomber and colleagues reported no DNA oxidative damage during Cu overload in *Escherichia coli* (Macomber et al., 2007). In our study, Cu down-regulated processes involving the nucleosome and DNA packaging complex, as inferred based on expression changes in histone proteins. Histone ubiquitination and ADP glycosylation have been reported to be related to DNA repair (Thorslund et al., 2015; Cao et al., 2016; Uckelmann and Sixma, 2017; Liszczak et al., 2018). However, in our western blot analysis, the major differences in ubiquitination were concentrated in proteins with a molecular weight higher than that of histone. Thus, further experiments are required to explore the effect of Cu stress on DNA.

The spatial distribution of ROS formation suggests that RNA is more likely to be damaged than DNA. We did not confirm the stability of RNA under Cu stress, but research in *E. coli* has shown that oxidative stress can disrupt translation and damage ribosomal RNA. When the 70S and 23S subunits, which harbor the peptidyl transferase center, were oxidized, cells lost translation activities related to ribosomal protein L12 (Willi et al., 2018). These results are consistent with our data, which showed that most of the ribosome subunits were down-regulated under Cu stress. Our functional annotation analysis also suggested that up-regulated rRNA biogenesis and U3 small nucleolar RNA-associated proteins may be related to the response to Cu stress.

According to our data, Cu stress down-regulated protein synthesis and up-regulated protein degradation in *C. neoformans*, while NAC prevented these changes, suggesting that disruption of protein homeostasis is the main effect of Cu-induced ROS. Regarding down-regulating protein synthesis, in *E. coli*, ribosomes are inhibited by gold nanoparticles, which prevent ATPase and tRNA binding (Cui et al., 2012). Additionally, the key regulatory role that ribosomes play in cell proliferation (Wilson, 2014), indicates that the down-regulation of the ribosomal subunits may be a key limiting factor in cell growth. Consistently, proteomic analysis of *Saccharomyces cerevisiae* showed that increased intracellular ROS suppressed global protein synthesis, which may be regulated by modulating the redox state of proteins (Topf et al., 2018). Furthermore, α subunit of eukaryotic initiation factor 2 (eIF2α) was identified to be the translational repressor in response to oxidative stress in *C. neoformans* (Leipheimer et al., 2019). These evidence of crosstalk between ROS and protein synthesis may help to elucidate the mechanism of Cu toxicity in *C. neoformans*.

Regarding the up-regulated protein degradation under Cu stress, the proteasome pathway is the principal route of intracellular protein degradation (Rousseau and Bertolotti, 2018). We showed that proteasome activity was increased during Cu stress and induced by ROS accumulation. The fact that the effect of Cu stress on growth restriction in *C. neoformans* was partially ameliorated by the proteasome inhibitor MG132 suggested that ubiquitin-mediated degradation underlies the growth restriction. The crosstalk between Cu or Cu-induced ROS and the proteasome pathway in *C. neoformans* remains unclear. However, clues can be found in several studies. The accumulation of ubiquitinated proteins alters the cellular redox state, leading to AMPK activation (Jiang et al., 2015). Additionally, cAMP/PKA can protect against oxidative damage (Geddes et al., 2016). Our study provides the perspective in that the inhibition of ubiquitin–proteasome pathway help to recover *C. neoformans* proliferation during high Cu or ROS stress. The protective function of MG132 may dues to the inhibition of degradation of key regulatory factors, such as heat shock factors. And the accumulation of abnormal proteins caused rapid increases in mRNA levels. This may induce the protective factors expression during recovery process (Bush et al., 1997; Lee and Goldberg, 1998; Mazroui et al., 2007).

Inhibition of protein degradation could not completely offset Cu toxicity. We identified changes in branched-chain amino acid biosynthesis, mitochondrial transmembrane transport, and ATP-binding cassettes in response to Cu stress, which may be associated with energy metabolism. Their relationship of these changes to Cu toxicity requires further study.

In summary, we assessed the global proteins response to Cu toxicity in *C. neoformans*. The core data may provide a theoretical basis for the development of antifungal drugs targeting the Cu ion metabolism pathway, to shorten treatment courses and increase therapeutic options. Continued attention should be paid to the characterization of the roles of specific proteins in virulence and the molecular mechanisms based on proteomic data.

## Data Availability Statement

The datasets presented in this study can be found in online repositories. The names of the repository/repositories and accession number(s) can be found below: ProteomeXchange Consortium (http://proteomecentral.proteomexchange.org) *via* the iProX partner repository with the dataset identifier PXD024098.

## Author Contributions

TS, YJL, YXL, HL, and YN performed the experiments. TS and CD designed the study. TS, YJL, HL, YG, and JW analyzed the data. TS, YX, CD provided equipment and funding. TS, YXL, and CD composed the manuscript. All authors contributed to the article and approved the submitted version.

## Funding

This research was supported by the National Natural Science Foundation of China (81801989 to TS), the Beijing Natural Science Foundation (5184037 to TS 7204288 to YXL), the Fundamental Research Funds for the Central Universities (3332018024 to TS), the China Postdoctoral Science Foundation (2021M693520 to HL), the National Natural Science Foundation of China (31870140 to CD), Liaoning Revitalization Talents Program (XLYC1807001), Beijing Key Laboratory for Mechanisms Research and Precision Diagnosis of Invasive Fungal Diseases.

## Conflict of Interest

The authors declare that the research was conducted in the absence of any commercial or financial relationships that could be construed as a potential conflict of interest.

## Publisher’s Note

All claims expressed in this article are solely those of the authors and do not necessarily represent those of their affiliated organizations, or those of the publisher, the editors and the reviewers. Any product that may be evaluated in this article, or claim that may be made by its manufacturer, is not guaranteed or endorsed by the publisher.

## Abbreviations

Cu, copper; LC-MS/MS, liquid chromatography-tandem mass spectrometry; NAC, N-acetylcysteine; ROS, reactive oxygen species.
