# Supplementary material for: Proteomic Analysis of Copper Toxicity in Human Fungal Pathogen Cryptococcus neoformans
Source: Front Cell Infect Microbiol. 2021 Aug 12;11:662404. doi: 10.3389/fcimb.2021.662404 (PMC8415117; doi:10.3389/fcimb.2021.662404)
Supplement: Supplementary file 1 [file DataSheet_1.docx]

**Supplemental legends**

**Supplemental Figure 1. Validation analysis of MS data based on parallel sample correlation analysis.**

1. **Coomassie Brilliant Blue staining of proteins isolated from *cmt1/2ΔΔ*.**
2. **Parallel sample correlation analysis.** Normalized intensity was subjected to the log2 transformation. Pearson phase relationship was used to assess the correlation. All R2 values of the parallel samples were close to 1, indicating satisfactory repeatability.

**Supplemental Figure 2.** [**Correlation**](C:/Users/tcyqj/AppData/Local/youdao/dict/Application/8.9.6.0/resultui/html/index.html#/javascript:;) [**analysis**](C:/Users/tcyqj/AppData/Local/youdao/dict/Application/8.9.6.0/resultui/html/index.html#/javascript:;) **of iTRAQ and PRM data based on differentially expressed ratio of target proteins.**

1. **Comparison between control and 0.5 mM Cu treatment groups.**
2. **Comparison between 0.5 mM Cu and 0.5 mM Cu plus 30 mM NAC treatment groups.**
3. **Comparison between control and 0.5 mM Cu plus 30 mM NAC treatment groups.**

**Supplemental Figure 3. Cell size and mophology of *C. neoformans* under MG132 treatment.**

1. **Cell mophology of *C. neoformans* obseved by 100 × microscope under 0.5 mM Cu, 10 μg/mL MG132 , 0.5 mM Cu plus 10 μg/mL MG132 treatment, respectively, after 4 or 24 hours shake cultivation.**
2. **Cell size of *C. neoformans* under 0.5 mM Cu, 10 μg/mL MG132 , 0.5 mM Cu plus 10 μg/mL MG132 treatment, respectively, after 4 or 24 hours shake cultivation (n=25).**

**Supplemental Figure 4. Expression level detected by MS of ROS associated proteins under Cu stress.**

**Supplemental Table 1. Primer pairs used in real-time PCR.**

**Supplemental Table 2.** **Quantitative proteomic analysis of *C. neoformans* in response to copper (Cu) toxicity.**

**Supplemental Table 3. Differentially expressed proteins in *C. neoformans* in response to copper (Cu) toxicity.**

1. **Comparative proteomic analysis of *C. neoformans* between Cu treated and untreated cells.**
2. **Comparative proteomic analysis of *C. neoformans* between Cu&NAC treated and Cu treated cells.**
3. **Comparative proteomic analysis of *C. neoformans* between Cu&NAC treated and untreated cells.**

**Supplemental Table 4. Parallel reaction monitoring (PRM) analysis of** **differentially expressed proteins identified in the proteomic analysis.**

1. **Quantitative analysis of representative peptides from target proteins.** One to four representative peptides were selected for each protein.
2. **Quantitative analysis of target proteins.** Three biological replicates were performed.

**Supplemental Table 5. Kyoto Encyclopedia of Genes and Genomes (KEGG) pathways enriched in response to copper (Cu) toxicity.**
