# Supplementary figures and images for: Proteomic Analysis of Copper Toxicity in Human Fungal Pathogen Cryptococcus neoformans
Source: Front Cell Infect Microbiol. 2021 Aug 12;11:662404. doi: 10.3389/fcimb.2021.662404 (PMC8415117; doi:10.3389/fcimb.2021.662404)

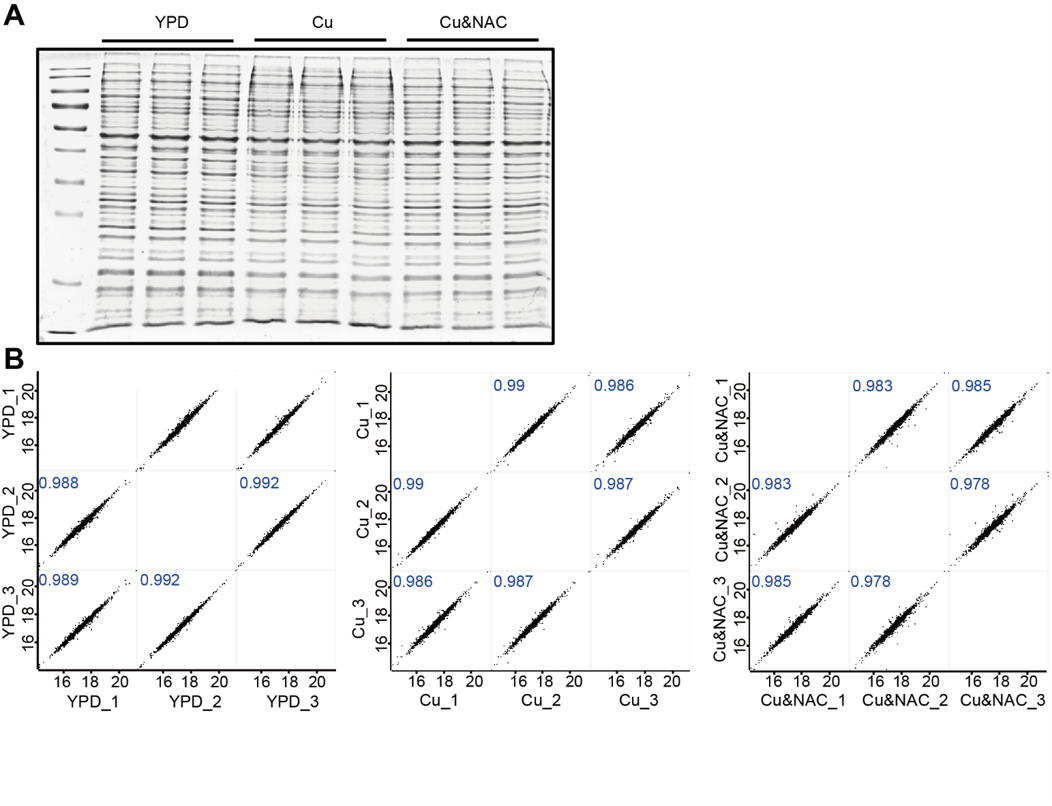

Supplement: Supplementary file 2 [file Image_1.tif]

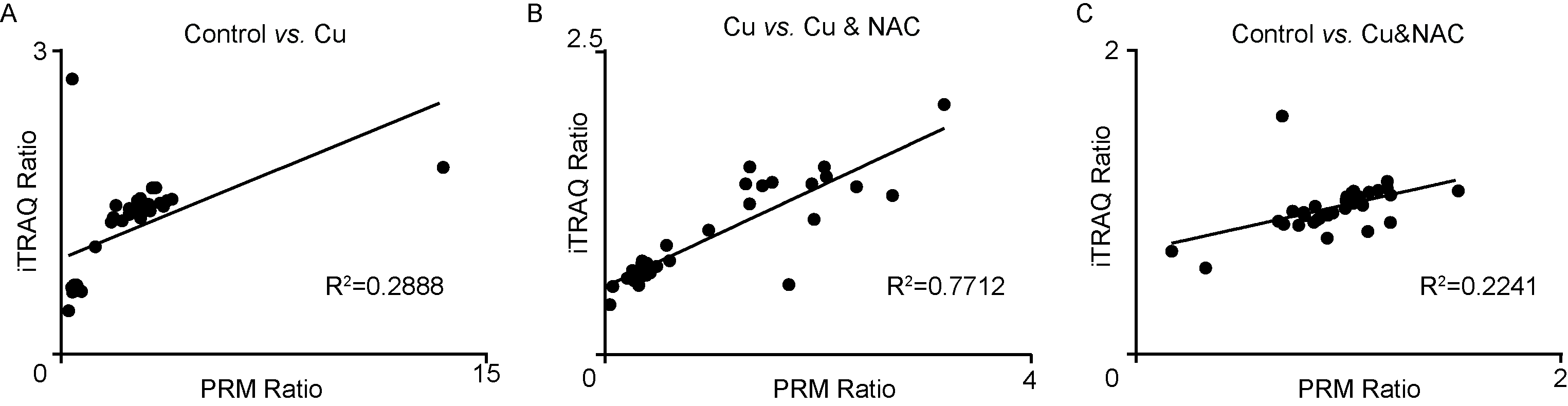

Supplement: Supplementary file 3 [file Image_2.tif]

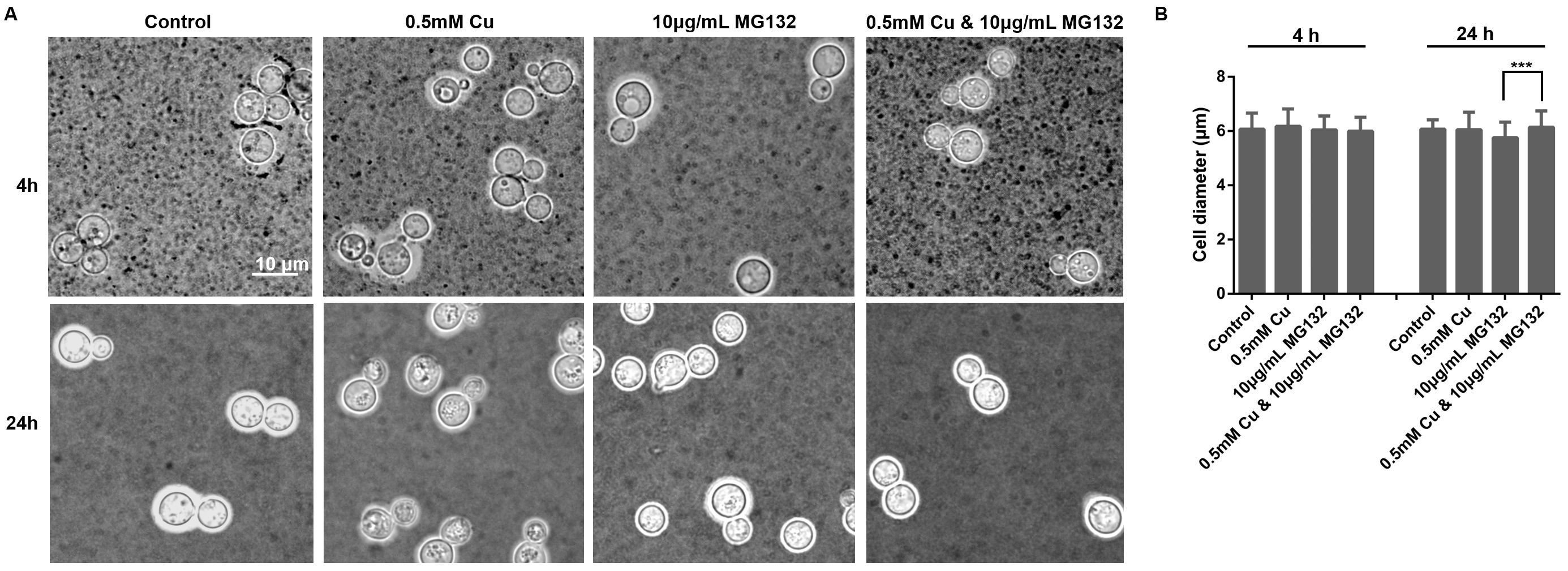

Supplement: Supplementary file 4 [file Image_3.tif]

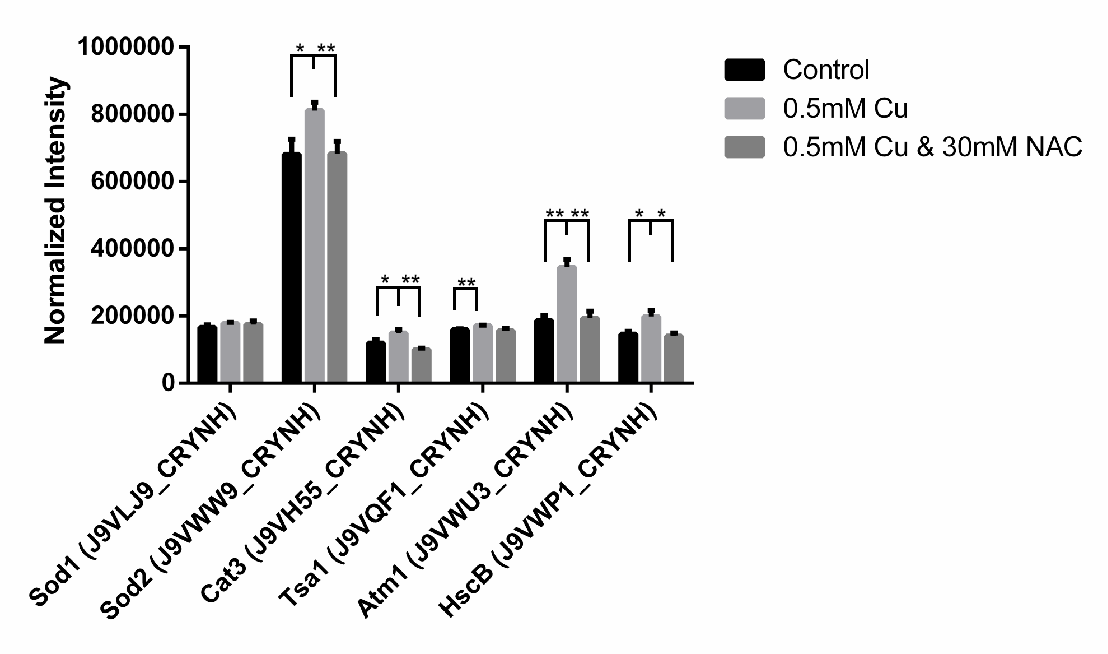

Supplement: Supplementary file 5 [file Image_4.tif]
